# Pathological Diagnosis of Hepatocellular Cellular Adenoma according to the Clinical Context

**DOI:** 10.1155/2013/253261

**Published:** 2013-04-18

**Authors:** Paulette Bioulac-Sage, Christine Sempoux, Laurent Possenti, Nora Frulio, Hervé Laumonier, Christophe Laurent, Laurence Chiche, Jean Frédéric Blanc, Jean Saric, Hervé Trillaud, Brigitte Le Bail, Charles Balabaud

**Affiliations:** ^1^Service d'Anatomie Pathologique, Hôpital Pellegrin, CHU Bordeaux, 33076 Bordeaux, France; ^2^U1053 Université Bordeaux 2, 33076 Bordeaux, France; ^3^Service d'Anatomie Pathologique, Cliniques Universitaires Saint Luc, Université Catholique de Louvain, 1200 Brussels, Belgium; ^4^Service d'Hépatologie, Gastroenterologie, Hôpital St André CHU Bordeaux, 33000 Bordeaux, France; ^5^Service de Radiologie, Hôpital St André CHU Bordeaux, 33000 Bordeaux, France; ^6^Service de Chirurgie Digestive, Hôpital St André CHU Bordeaux, 33000 Bordeaux, France; ^7^Service Hépatobiliare et Pancréatique, Hôpital Haut Lévêque CHU Bordeaux, 33604 Pessac, France

## Abstract

In Europe and North America, hepatocellular adenomas (HCA) occur, classically, in middle-aged woman taking oral contraceptives. Twenty percent of women, however, are not exposed to oral contraceptives; HCA can more rarely occur in men, children, and women over 65 years. HCA have been observed in many pathological conditions such as glycogenosis, familial adenomatous polyposis, MODY3, after male hormone administration, and in vascular diseases. Obesity is frequent particularly in inflammatory HCA. The background liver is often normal, but steatosis is a frequent finding particularly in inflammatory HCA. The diagnosis of HCA is more difficult when the background liver is fibrotic, notably in vascular diseases. HCA can be solitary, or multiple or in great number (adenomatosis). When nodules are multiple, they are usually of the same subtype. HNF1**α**-inactivated HCA occur almost exclusively in woman. The most important point of the classification is the identification of **β**-catenin mutated HCA, a strong argument to identify patients at risk of malignant transformation. Some HCA already present criteria indicating malignant transformation. When the whole nodule is a hepatocellular carcinoma, it is extremely difficult to prove that it is the consequence of a former HCA. It is occasionally difficult to identify HCA remodeled by necrosis or hemorrhage.

## 1. Introduction 

The diagnosis of hepatocellular adenomas (HCA) may occasionally be difficult for the following reasons.

- The nodule is discovered in a context different to what we are used to see, such as in men, in women not exposed to oral contraceptives (OC), in older persons, or in children.
- The tumor *per se* may be difficult to identify due to the partial necrosis or to the major remodeling of the tumor leading to the presence of criteria seen mostly in focal nodular hyperplasia (FNH) and/or to difficulties in differentiation from hepatocellular carcinoma (HCC).
- The presence of an underlying liver disease such as nonalcoholic steatohepatitis (NASH), vascular disorder, and fibrosis.
- HCA, HCC, and FNH or different HCA subtypes can be present in the same patient, some more prone to HCC transformation, with the difficult task, in some cases, to differentiate HCA from HCC.
- Finally, HCA can be discovered unexpectedly in patients treated for other liver tumors or developed in the context of diseases affecting the liver or other organs.

In this paper, we review the clinical/epidemiological context of HCA, based on our experience (personal cases and consult cases); all these cases being classified according to the pathomolecular classification into four groups, as previously published are HNF1*α*-inactivated HCA (H-HCA), inflammatory HCA (IHCA), *β*-catenin activated HCA (b-HCA and b-IHCA) and unclassified HCA (UHCA).

## 2. HCA: Age, Gender, and Oral Contraceptives

(1) The great majority of HCA occurs in middle age women genitally active, taking OC (at least in many Northern European countries and in North America) [1]. The magnitude of the risk of HCA in OC users is yet defined but is considered to be dose and time dependent. The difference in the incidence of HCA between countries among women taking OC has not been evaluated but seems real. Our experience is presented in Table 1. The incidence seems higher in France than in the US. A possible explanation could be the youngest age of the women exposed to the contraceptive pills in France compared to the US. Less than 20% of women with HCA are not exposed to OC or are exposed for very short periods of time.

If the role of OC in the development of HCA is certain, the individual susceptibility to the risk is of paramount importance. No particular HCA subtypes have been observed in women; however, HCA in very young women (in their twenties) taking or not OC without specific etiology are rare and found, in our experience, mainly in the b-HCA and UHCA subgroups.

(2) HCA in men are rare [2–8]. To the exception of HCA cases related to MODY3 (H-HCA) and male hormone administration (b-HCA), the immense majority are found in the IHCA and b-IHCA subgroups. They are usually solitary. Patients are often overweight with one or several features of the metabolic syndrome. As the risk of HCC is important, resection of the nodule is recommended even for nodules smaller than 5 cm [9, 10].

(3) HCA occurring in infants, adolescents, or young adults are mainly related to specific etiology such as vascular disorders, familial adenomatous polyposis (FAP), or MODY 3. 

(4) HCA in patients over 65 are rare. We have observed 4 H-HCA cases, all in women. 

(5) In addition to age, gender, OC, and background liver diseases (see below), the biological and radiological parameters are also good indicators of HCA subgroups. Radiologists are now able to identify with confidence typical H-HCA and IHCA. Furthermore, a raised CRP level in the blood is a strong argument to identify IHCA. 

## 3. HCA and HCC

It is well admitted that HCA may transform into HCC [11–25]. However, the risk of malignant transformation of HCA cannot be reliably quantified yet. Several series are concordant to show that approximately 5% of patients, whom HCA has been resected, had pathological evidence of HCC within their HCA. This figure, however, does not take into account fully transformed HCA where evidence of the preexisting benign lesion might have disappeared. The risk of malignant transformation is correlated with the b-catenin mutated subtype, and with the size of the HCA. HCA malignant transformation is unusual for nodules <5 cm. These results suggest that small HCA occurring in women could be safely observed, as they are also at low risk of bleeding. 

HCC that developed on HCA are typically well differentiated without vascular extension or satellite nodules. AFP measurement is not reliable as it is usually normal. The prognosis is—compared to HCC in cirrhotic patients—relatively good if we consider that tumors are usually large tumors (>5 cm).

Prevalence of malignancy within HCA is 10 times more frequent in men than in women and management of HCA should primarily be based on gender. In addition to men, reported cases of HCC on HCA concern rare etiologies such as glycogenosis, male hormone administration, and vascular diseases. Metabolic syndrome also appears as an emerging condition associated with malignant transformation of HCA particularly in men and is the likely most frequent predisposing condition for HCC in this setting. For the pathologists, there are different degrees of difficulties to make the diagnosis of HCA transformation into HCC (see article Balabaud et al. in this issue). 

**Figure 1:**
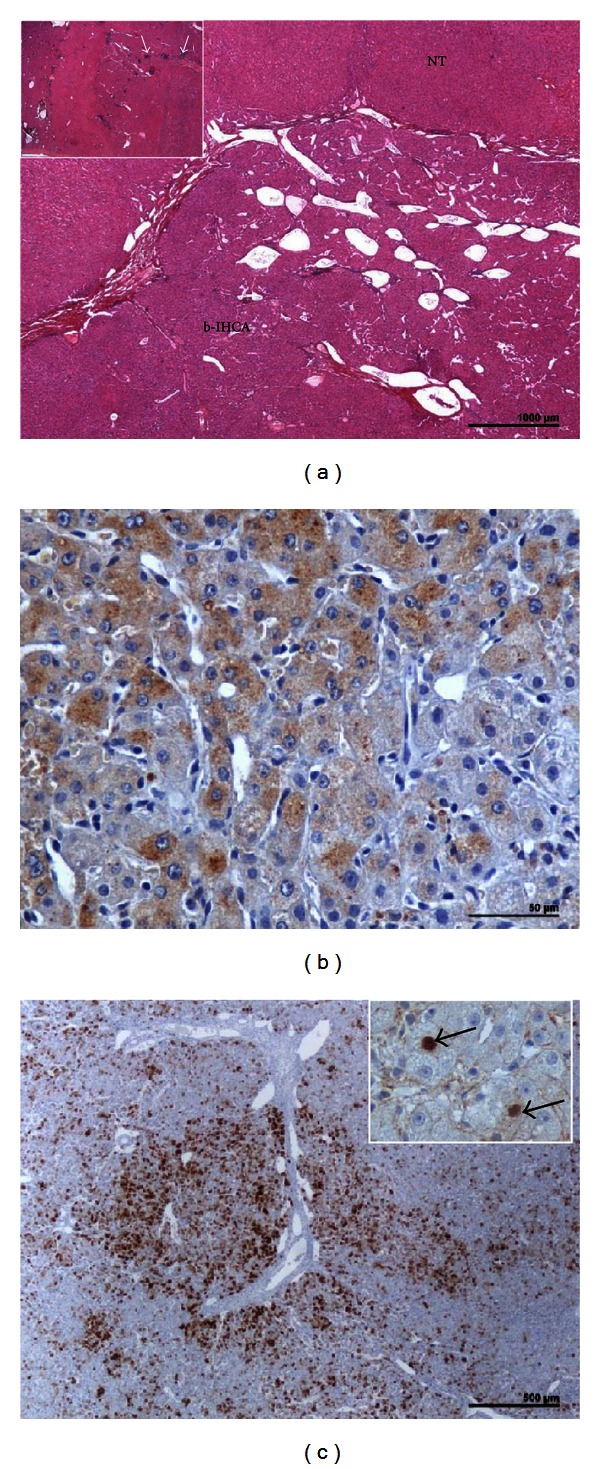
b-IHCA (with discovery of an HCC 12 years later). A woman born in 1959. Discovery of a nodule 17 cm. Oral contraceptives 4 years, BMI 21.5. Surgery 1984: right hepatectomy but incomplete resection. Followup refused by the patient: 2 pregnancies. Patient seen 12 years later with numerous liver nodules of a well-differentiated HCC. Death occurred few months later. (a) H&E—proliferation of hepatocytes with some atypia (not visible at this magnification) and numerous dilated vessels; no arguments for overt malignancy; inset: a few inflammatory infiltrates (arrows). (b) Moderate expression of SAA by adenomatous hepatocytes. (c) Heterogenous, patchy expression of GS; inset: aberrant expression of a few hepatocytic nuclei (arrows).

(1) The tumor is definitively an HCC: malignant transformation of an HCA is likely when the HCC occurs in a specific context such as glycogenosis, male hormone administration, or when the diagnosis of HCA has been established several years before. The link exists but cannot be demonstrated when the cause of the putative HCA is not well documented (i.e., in patient exposed to anticonvulsive drugs), or when there are areas in the tumor, particularly at the border, looking benign but that could correspond to a very well-differentiated HCC. 

(2) The tumor is benign but there are foci with cytological/architectural criteria in favor of premalignant changes (i.e., rosette formation, increased CD34 staining, irregular/decreased reticulin network).

(3) The tumor is possibly malignant, at least in part (i.e., areas with loss of reticulin, diffuse CD34 staining positivity, GPC3 even mild and focal, etc.). In many occasions, the diagnosis of true malignancy remains impossible to assert and the term “borderline tumor HCA/HCC” can be used. In our own series, we observed 17 cases of HCC possibly linked to HCA with 2 deaths (Figure 1).

## 4. HCA Occurring in the Context of Specific Etiology

### 4.1. Vascular Diseases

Many different types of hepatocellular nodules ranging from nodular regenerative hyperplasia (NRH), focal nodular hyperplasia (FNH), and macroregenerative nodule (MRN)/FNH-like, to HCA and HCC have been described in different types of vascular disorders [26–42] such as Budd Chiari syndrome [26–31], hereditary hemorrhagic telangiectasia [32], agenesis of the portal vein, intrahepatic shunts (congenital or acquired) [33–40], and the Fontan procedure [41, 42]. Unfortunately there is today no reliable data using modern techniques of identification of these nodules (imaging, histopathology, molecular biology). Recent data suggest that the majority of nodules are MRN/FNH-like and in addition, different HCA subtypes have been observed with possible malignant transformation [31]. In our personal experience (including consults), we have observed 4 cases (2 with HCC).

### 4.2. HCA and Genetic Disorders

#### 4.2.1. Glycogenosis

 In a large series of 43 patients published in 1997, 51.8% of patients with type 1 and 25% of patients with type 3 glycogen storage disease had HCA at the time of the study. The male to female ratio was 2 to 1 in type 1, and no female had adenomas in type 3 [43]. In a retrospective chart review performed in 117 patients with glycogenosis 1a, it was shown that metabolic control measured on the basis of serum triglyceride concentration may be related to HCA formation [44]. Immunohistochemistry (IHC) has been described in 2 large series of type 1 glycogenosis [45, 46]. IHCA was the main subtype; b-HCA has been also observed but not H-HCA [46]. In our series, we observed in 2 cases different HCA subtypes: IHCA, b-HCA, and b-IHCA. One male patient with type 3 glycogenosis and hepatic nodules detected at the age of 3 died at the age of 27 of HCC in our unit; the diagnosis of HCA and HCC was based on radiological criteria. HCC is a rare but major complication of glycogenosis [17].

#### 4.2.2. Familial Adenomatous Polyposis

**Figure 2:**
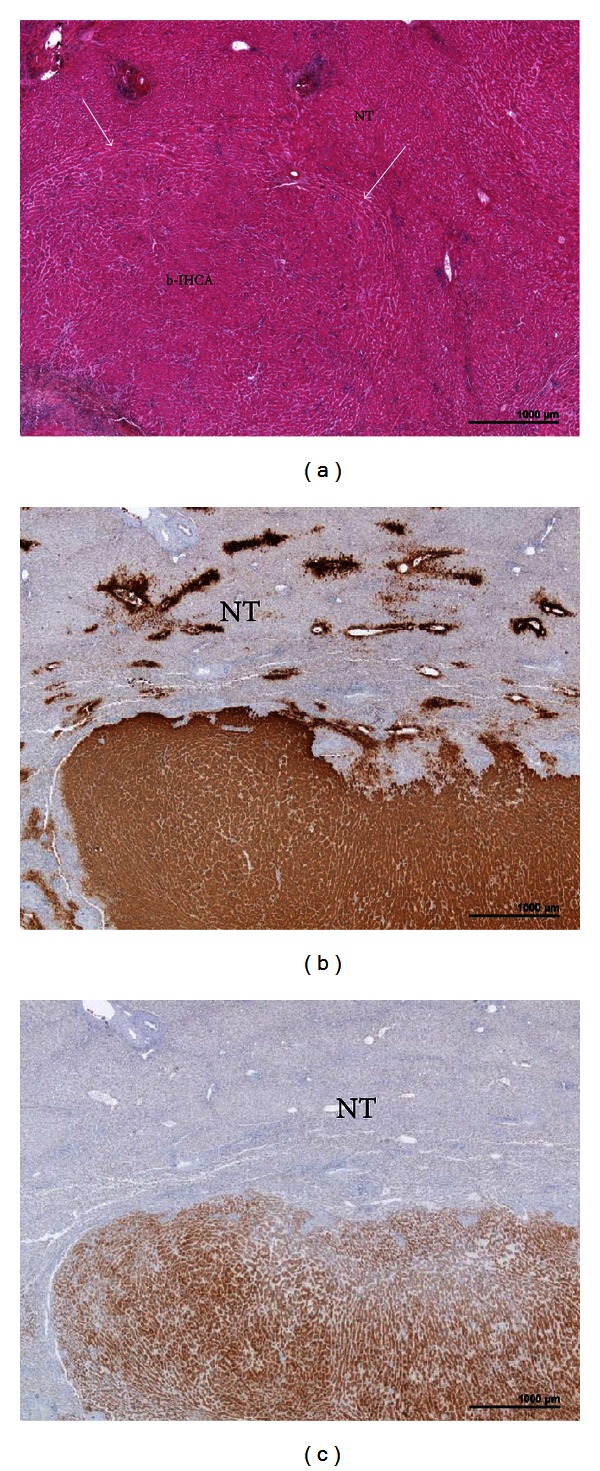
b-IHCA in the context of a FAP. A man born in 1966. Familial adenomatous polyposis (surgery in 1990 and 1996). Liver nodule discovered by chance, 9 cm. BMI 32.3. Segmentectomies V and VI. (a) H&E: HCA with ill-defined border (arrows). (b) Strong and diffuse expression of GS, contrasting with normal GS staining limited to a few centrolobular hepatocytes in the nontumoral liver (NT). (c) Diffuse expression of SAA in adenomatous hepatocytes with sharp demarcation from the surrounding NT.

 HCA in patients with FAP have been reported previously [15, 47–50] with inactivation of HNF1*α* [48] as well as biallelic inactivation of the APC gene [49, 50]. Malignant transformation has also been described [15]. In our series we have observed 2 cases of b-IHCA associated with FAP (Figure 2).

#### 4.2.3. MODY3

 The discovery of H-HCA in MODY3 is a great success of molecular biology with important clinical consequences [51–55]. The diagnosis of MODY3 should be evoked in H-HCA in the following circumstances: young age of the patient, adenomatosis, history of familial HCA, and diabetes in young age. H-HCA in men are observed only in MODY3 patients. We have confirmed the diagnosis of H-HCA due to MODY3 in 2 families [53, 55].

#### 4.2.4. Tyrosinemia

 Cases of HCA have been reported in tyrosinemia [1]. The diagnosis of HCA, in cirrhotic patients, remains very difficult to establish.

### 4.3. Drugs

HCA and HCC have been reported in patients taking male hormones for medical purposes, that is, Danazol [56–59], or to increase their muscular mass such as body builders [60, 61]. We had at least 2 cases of women taking OC and exposed also to Danazol; both were b-HCA. In one case there were multiple nodules, the largest one shrunk massively after stopping Danazol and all nodules presented features of involute HCC. The link between HCA and the long-term exposition to antiepileptic drugs is not well established [62–66]. We saw 3 such cases possibly linked to antiepileptic drugs. 

### 4.4. Overweight/Obesity

**Figure 3:**
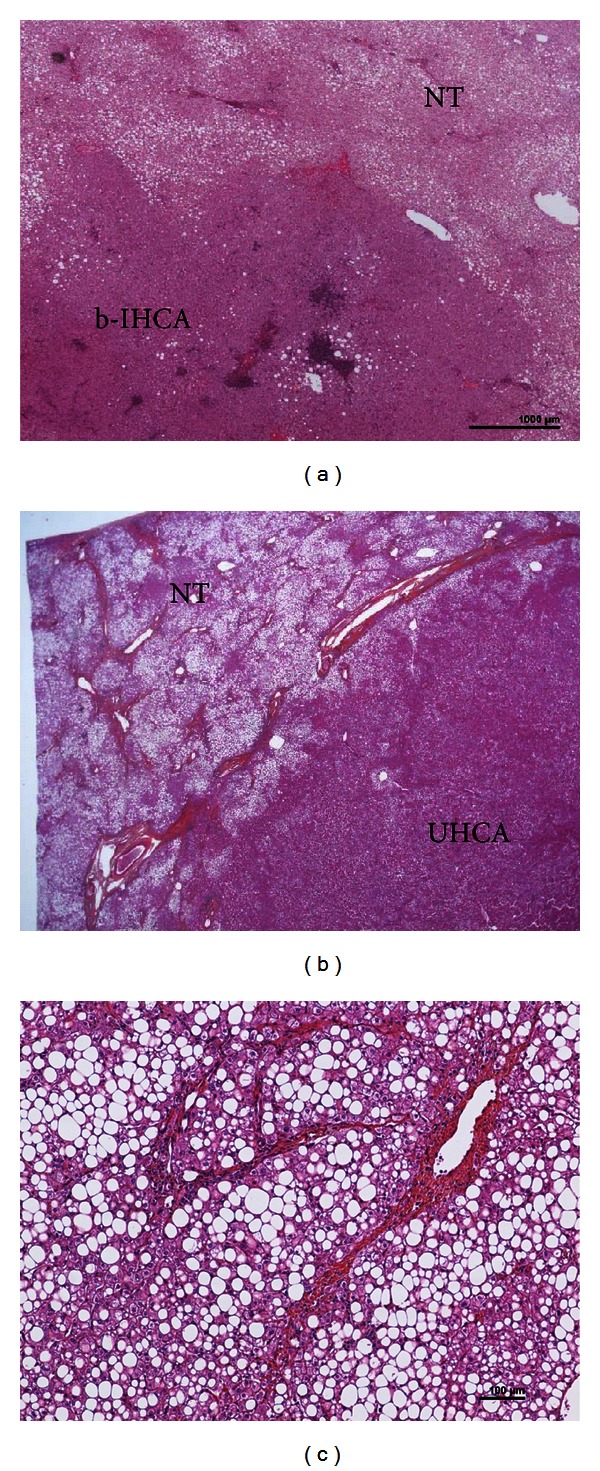
HCA on the background of NASH. (a) A woman born in 1956. Several nodules in the liver, largest 8 cm. Oral contraceptives for 31 years; BMI 24.6. Surgery in 2007 (several tumorectomies and segmentectomies). H&E: IHCA (typical expression of SAA and CRP—not shown), very mild steatosis, contrasting with highly steatotic (60%) nontumoral liver (NT): NASH with mild activity, without fibrosis. (b-c) Woman born in 1973, metabolic syndrome (noninsulin-dependant diabetes, hypercholesterolemia, hypertriglyceridemia). Oral contraceptives 15 years, BMI 31.6. Two liver nodules, largest 26 mm in segment IV. Segmentectomy IVB plus radio frequency of the other nodule (1 cm) in segment VIII. (b) H&E: nonsteatotic HCA (without immunohistochemical characteristics), classified as UHCA; its limit contrast with severe steatotic non tumoral liver (NT). (c) H&E: nontumoral liver: NASH with severe steatosis (80%), mild activity, and septal fibrosis (stage 3).

The number of HCA noticeably increased faster in the 2001–2011 period compared to the 1990–2000 period. This phenomenon concurred with an increasing number of patients overweight or obese [67–69]. More overweight patients are found to harbor IHCA than H-HCA. Females still represent the great majority of overweight/obese patients with HCA. Overweight/obese male or female patients constitute a new entity in the IHCA and *β*-catenin activated IHCA subgroups. Overweight/obesity may soon represent a major risk of malignant transformation of HCA, possibly because of the activation of the IL-6 pathway. In HCA, the nontumoral liver is usually normal or subnormal. Steatosis (mild to severe) is quite often observed in overweight/obese patients with or without metabolic syndrome; NASH is rare [70]. Most of the time, there is a clear difference on gross pathology or under the microscope between the tumoral and nontumoral liver (Figure 3). The distinction may be difficult at first glance when the nontumoral liver is steatotic as well as the tumor. Steatosis in the nontumoral liver can also be observed in other instances such as in alcoholic liver disease, glycogenosis, or the Fanconi anemia.

### 4.5. Anemia

(1)* Fanconi anemia.* The Fanconi anemia is an autosomal recessive disease, causing secondary aplastic anemia and congenital abnormalities, associated with an increased risk of tumors [56, 71, 72]. In patients with the Fanconi anemia, androgen therapy and iron overload may contribute to the development of HCA and HCC; the latter may occur as a transformation of HCA. With prolongation of survival, continued development of liver tumors can be expected. Routine detection should therefore be considered in these patients. 

(2)* beta thalassemia*. Hepatocellular adenoma has been reported [1].

(3)* Anemia of chronic disease*. In 2002, severe anemia of chronic disease was described in an unusual group of patients with glycogen storage disease type 1a. The anemia was directly related to the presence of large hepatic adenomas that inappropriately produced hepcidin [73, 74]. A similar mechanism was described in another case not related to glycogenosis [75]. In our experience at least 2 patients were investigated during several years for the diagnosis of inflammatory anemias without any clues until one or several liver nodules were discovered. The anemia was cured after resection of the IHCA [76]. In patients with IHCA, it is not rare to observe biological criteria of inflammatory anemia [10]. 

### 4.6. Endocrine Disorders

(1)* Polycystic ovary syndrome.* A case of a young woman with HCA in a context of polycystic ovary syndrome, associated with high levels of androgens and following a high dose hormonal therapy, has been described [77]. We observed a similar case (IHCA).

(2)* Cushing's syndrome.* To our knowledge no case of HCA has been reported yet in Cushing syndrome patients. In our file, one b-HCA occurring in a patient with a Cushing syndrome became malignant years later; however, this old case was poorly documented.

### 4.7. Fibrotic Background Liver

In severe fibrosis/cirrhosis, the diagnosis of HCA was not reported until recently in alcoholic patients [78]. Here the difficulty is to differentiate an IHCA from a MRN/FNH-like expressing inflammatory proteins. In our experience MRN and MRN-FNH-like, which are frequently observed in cirrhotic background, can express CRP. Molecular data are necessary to identify with certainty HCA in cirrhotic patients. 

## 5. Association of HCA with Other Tumors

### 5.1. Association of HCA with HCC and Nonhepatocellular Tumors

**Figure 4:**
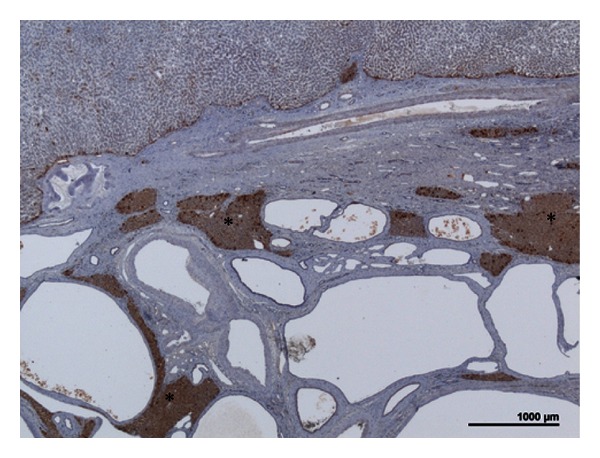
H-HCA associated with polycystic disease. Woman born in 1954. In 1991: kidney transplantation for polycystic kidney disease. In 2000: left hepatectomy for a 11 cm liver nodule. On oral contraceptives for 2 years, BMI 21.2. LFABP immunostaining: numerous biliary cysts with some areas of liver normally expressing LFABP (asterisk), contrasting with a portion of the H-HCA (upper) where LFABP is sharply decreased.

We have observed HCA associated with other tumors occurring elsewhere in the liver. In all these cases, HCA were in the H-HCA subgroup, associated with an HCC, an angiomyolipoma, and a mucinous cystadenoma, and one was associated with cysts in the context of a kidney polycystic disease (Figure 4). These associations are possibly fortuitous in the 2 first observations. We cannot, however, rule out the possibility of a common genetic parameter, at least in the last observation. 

### 5.2. Association between Different Benign Hepatocellular Tumors

**Figure 5:**
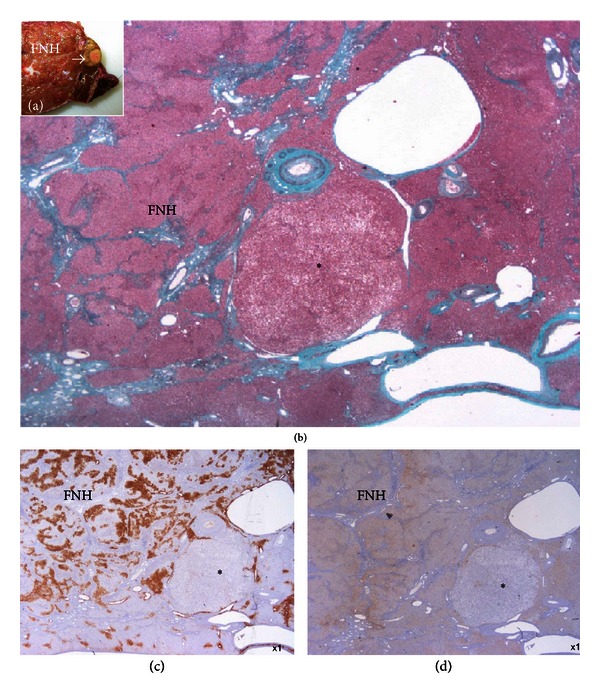
FNH associated with H-HCA. Woman born in 1966. Three FNH, largest 10 cm. Surgery in 2008 (segments V and VI). Discovery on the resected specimen of several small H-HCA. (a) Fresh specimen: typical FNH closed to a small yellow nodule (arrow). (b) Masson's trichrome: typical aspect of FNH, nearby a small steatotic nodule (asterisk). (c) GS staining is negative in the nodule, contrasting with map-like positivity in FNH. (d) LFABP is lacking in the small nodule, whereas it is normally expressed in FNH (as in nontumoral liver, not shown).

**Figure 6:**
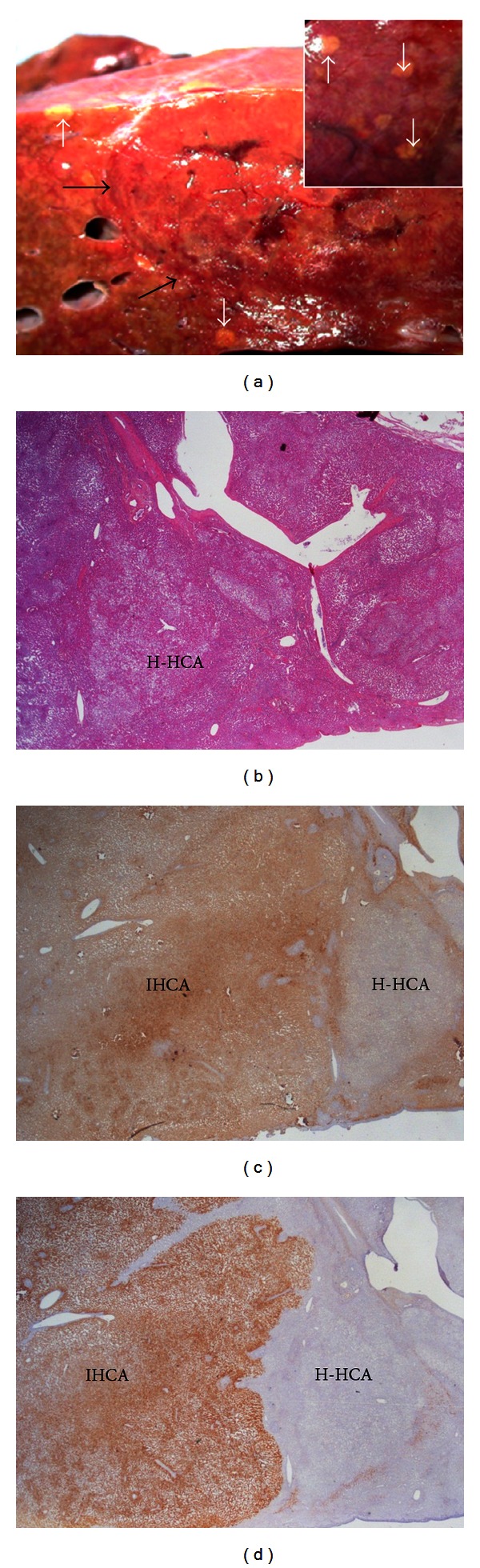
H-HCA and IHCA. Woman born in 1965. Abnormal liver function tests. Hypertriglyceridemia. Multiple nodules largest 9.5 cm. BMI 21.5. 18-year oral contraceptives. Left hepatectomy in 2011. (a) Fresh specimen—the large reddish nodule is not well limited (black arrows), associated with numerous yellowish small nodules in the surrounding resected parenchyma, sometimes visible under the Glisson's capsule (white arrows). (b) The small nodules are steatotic. (c) LFABP is lacking in all the small nodules (H-HCA), contrasting with normal expression in the nearby IHCA. (d) CRP is strongly expressed in IHCA, whereas it is negative in the small nodule of H-HCA.

-  
*Association of HCA and FNH.* The association is probably not fortuitous. FNH are particularly frequent in adenomatosis, and it is not rare that small H-HCA could be fortutiously discovered on the resected specimen (Figure 5). It is well known that liver vascular diseases are prone to the development of FNH. As such they may also play a role in the development of HCA [79].
-  
*Association of different HCA subtypes.* H-HCA and IHCA are rarely associated in the same liver; we have observed, however, a few cases presenting such association (Figure 6). This association supports the concept of an individual susceptibility to develop HCA, common to several subtypes.
-  
*Association of IHCA and b-IHCA.* In cases with multiple IHCA, some nodules can be in addition b-catenin activated indicating that b-catenin mutation could be a late event in the development of the nodule; indeed, in such cases large nodules were b-IHCA whereas the small ones were IHCA.

### 5.3. Association of HCA with Extra Hepatic Tumors

(1) In 1989, Wanless et al. reported 13 cases with multiple FNH associated with other lesions such as hemangioma of liver meningioma, astrocytoma, telangiectasia of the brain, berry aneurysm, dysplastic systemic arteries, and portal vein atresia [80]. In the same paper, Wanless described the so-called “telangiectatic subtype of FNH” which occurs in this syndrome as well as in a minority of patients with solitary FNH. Today we know that the so-called telangiectatic FNH are IHCA [81, 82]. FNH with major sinusoidal dilatation, however, does exist [83]. We still have to clarify the association between the above brain tumors and benign hepatocellular nodules. 

(2) HCA are so rare that patients with cancer (breast, colon, etc.) and a nodule in the liver are thought to have, *a priori*, a metastasis. We have encountered 3 patients already treated by chemotherapy where HCA were discovered by chance during the followup.

## 6. HCA with Rare or Misleading Features

### 6.1. Rare Findings in HCA

**Figure 7:**
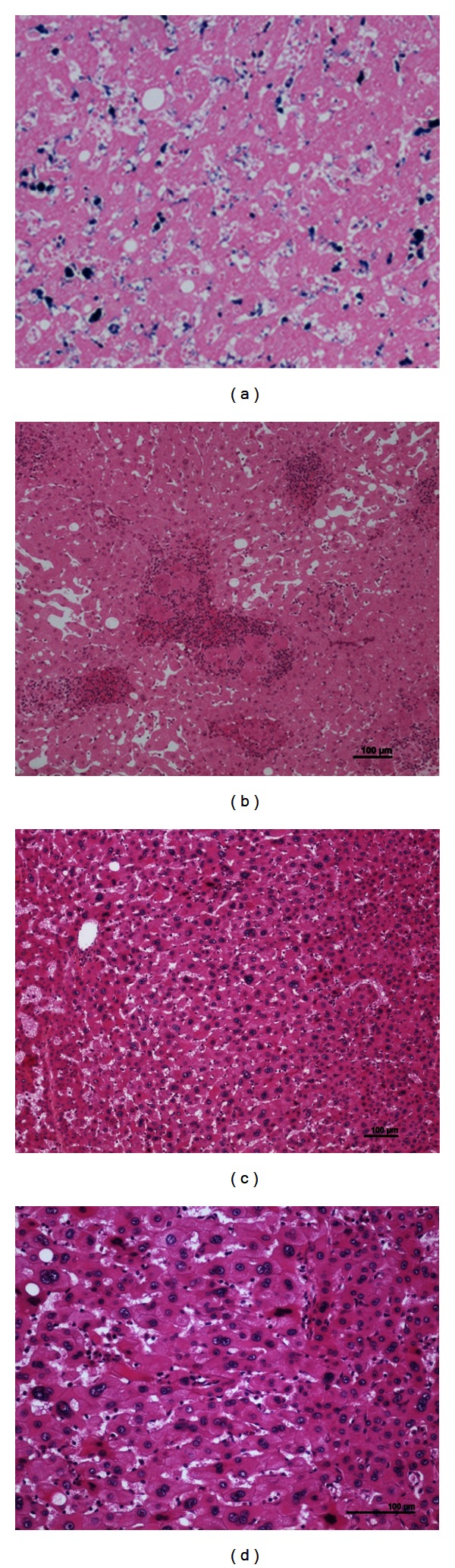
Rare, misleading cytological findings. (a) Woman born in 1964. Abnormal liver tests and anemia. No oral contraceptives, BMI 40.4. Alcohol (30 to 40 g per day), hypercholesterolemia, and type 2 diabetes. Biopsy: IHCA. Right hepatectomy 2006: IHCA. The anemia corrected several months after surgery. Perls staining: positivity in sinusoidal cells of the IHCA. (b) Woman born in 1936. Oral contraceptives 21 years. Several liver nodules. Tumorectomies in 1989. H&E: numerous epithelioid granulomas are widespread within the IHCA. (c-d): Woman born in 1947. Massive bleeding. No oral contraceptives, BMI 21.5. Right hepatectomy 2000. Massive liver necrosis. Pathological diagnosis: IHCA. H&E: areas with cytological abnormalities: dystrophic nuclei (irregular, hyperchromatic), nearby necrotic/hemorrhagic zones of the IHCA.

Iron [74] or other pigments such as lipofuscin granules, Dubin Johnson pigment [7, 8, 16, 84], hematopoiesis, calcifications [85], and inflammatory granulomas [86–88] are occasionally observed in HCA. Iron is located in hepatocytes in the Fanconi anemia. Surprisingly no iron was observed in HCA with chronic anemia related to hepcidin production. In our experience, among our cases of HCA associated with chronic anemia, we did not observe iron overload except in 2 cases where iron was almost exclusively found in Kupffer cells (Figure 7(a)). 

### 6.2. Cytological/Architectural Abnormalities Not Linked to Malignancy Transformation

Abnormalities such as mainly dystrophic nuclei or 2-3 cells thick hepatocytic plates are often seen when HCA exhibits large hemorrhagic/necrotic areas (Figure 7(b)). These misleading features are probably linked to a secondary regenerative process.

### 6.3. HCA Presenting Features of FNH

**Figure 8:**
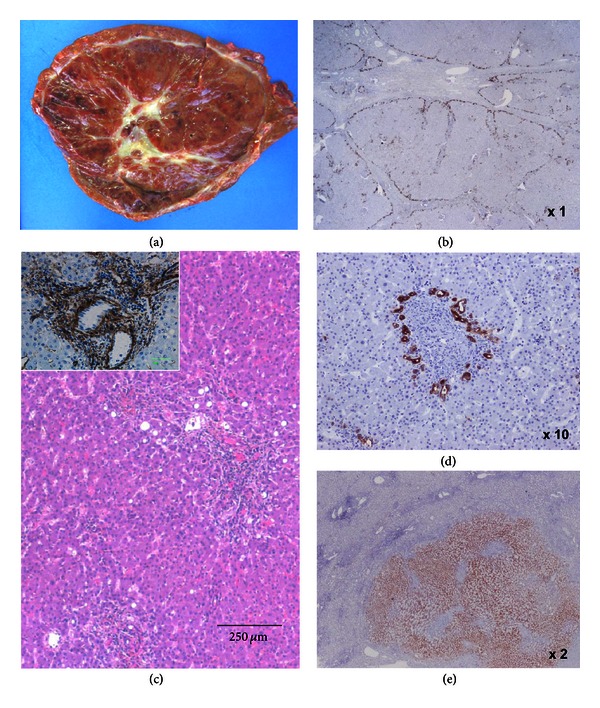
HCA looking like FNH. Same as patient 7A. (a) Fresh specimen: large subcapsular reddish tumor, with a white stellate area of loose fibrosis in the center. (b) CK7 is expressed at the border of fibrotic bands delineating nodules. (c) H&E (*α*SMA insert). The overall aspect is in favor of an IHCA. (d) Ductular reaction around inflammatory pseudo-portal tracts. (e) CRP is diffusely expressed in the nodule. The diagnosis of IHCA was reinforced by the disappearance of the chronic anemia after resection of the nodule.

HCA can also be remodeled following necrosis/hemorrhage. Fibrotic bands and even scars can form so that HCA look like FNH; these changes are more frequently seen in IHCA subtypes (Figure 8). The arguments to favor one diagnosis over the other rely mainly on clinical, biological, and IHC data; this may not represent, however, strong enough arguments in some difficult cases. 

### 6.4. HCA Number and Size Variation

**Figure 9:**
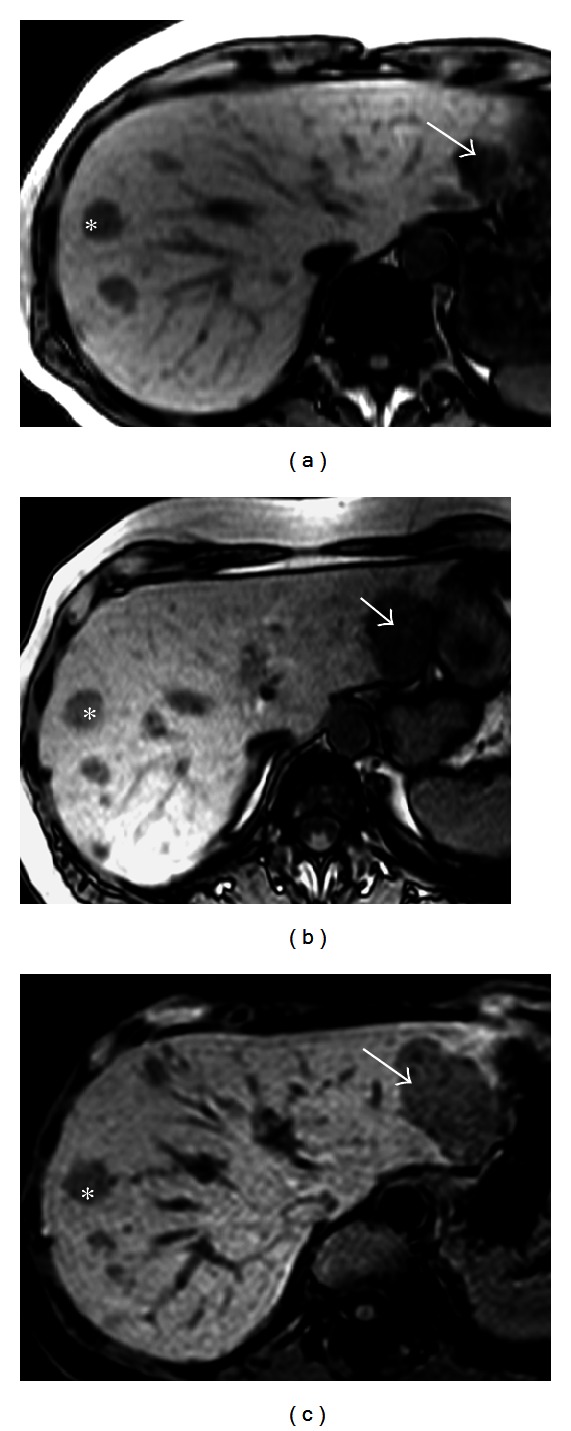
H-HCA size increment. Woman born in 1952. Abnormal liver function tests. Oral contraceptives 13 years, BMI 25.4 MRI adenomatosis, largest nodule 6 cm. Segmentectomies IV and VI 2001. Follow-up: increase size of one nodule Segmentectomy III, 2010. (a–c) Phased-opposed T1 weighted MR images in 2001 (a), 2007 (b), and 2010 (c) showing multiple hepatocellular adenomas showing hypointense because of the massive fat component. The nodule located in segment II (arrow) gradually increases in diameter between 2001 and 2010. As the 13 mm nodule located in segment VIII (asterisk), other nodules did not change their size.

HCA are solitary or multiple (from few to many). Many HCA defined the so-called entity adenomatosis (arbitrarily more than 10 nodules). For surgeons, adenomatosis is defined also by the number of nodules whose size raises therapeutic decision. Therefore, we have to distinguish cases with a single large nodule accompanied by myriads of small millimetric nodules from cases with multiple large nodules. Adenomatosis is more frequently observed in H-HCA and to a lesser extent in IHCA [10]. Apart from metabolic disorders, adenomatosis is rare in b-HCA and UHCA. Interestingly enough in the immense majority of cases, HCA when detected seems to be already at their maximum size (for those not resected). During followup (and after OC stopped), size remains stable or decrease. The impression, that needs to be confirmed, is that H-HCA remains stable and that IHCA tends to decrease. It is necessary to remember that in HNF1*α*-inactivated adenomatosis, there are myriads of small HCA that tend to aggregates to form larger nodules [89]. We have observed 2 HNF1*α* adenomatosis where such large nodules were interpreted as increasing in size, requiring reintervention (Figure 9). It is not known if the hemorrhagic risk is linked to the apparent global size of the nodule or not.

## 7. Recommendations and Conclusion

If it is tempting to publish rare cases, such as HCA with unusual cytological abnormalities, HCA in rare pathological context (beta thalassemia, tyrosinemia, etc.), HCA associated with rare hepatic or extrahepatic tumors, HCA have to be classified in subgroups with the help of IHC, to understand the pathophysiology of the disease and, in the long term, to better diagnose liver tumors and adapt the management of the patients. Not all HCA can be correctly classified with histological tools only. To prevent this limitation, it is recommended to freeze tissue, in order to perform molecular studies, that are particularly important to understand unusual and unclassified HCA cases. It is hoped that the pathomolecular classification of hepatocellular adenomas developed on normal as well as on fibrotic liver will help us to make progress in this field.

## Figures and Tables

**Table 1:** Hepatocellular adenomas: Bordeaux cases.

	Total	H-HCA	IHCA	b-IHCA	b-HCA	UHCA
*n*	184*	66	68	13	14	22
Mean age (extreme)	40 (14–66)	41 (14–65)	41 (25–59)	35 (18–59)	35 (14–66)	36.5 (22–52)
*n* W	163	62	59	8	11	22
Mean age (extreme)	40 (21–66)	41 (23–60)	40 (25–54)	35.5 (26–46)	35 (21–66)	36.5 (22–52)
*n* W (OC)	146	52	54	7	11	21
*n* W BMI > 25	52	13	24	0	2	12
*n* M	19	3**	9	5	2	0

*n*: number; W: adult women; M: adult men; BMI: body mass index; OC: oral contraceptives; *includes 2 children; **2 patients with MODY3.
